# Bovine Lactoferrin Compared With Ferrous sulfate for Treating Iron-Deficiency Anemia in Bangladeshi Women—A Randomized Controlled Trial

**DOI:** 10.1016/j.tjnut.2026.101672

**Published:** 2026-06-17

**Authors:** Tanvir M Huda, Sajia Islam, Nazia Binte Ali, Shams E Tabriz Bhuiyan, Qazi Sadequr Rahman, Rubhana Raqib, Armando Teixeira-Pinto, William Tarnow-Mordi, Shams El Arifeen, Michael J Dibley

**Affiliations:** 1 Sydney School of Public Health, Faculty of Medicine and Health, The University of Sydney, Sydney, NSW, Australia; 2 Maternal and Child Health Division, International Centre for Diarrhoeal Disease Research, ICDDR, B, Dhaka, Bangladesh; 3 Department of Global Health, The Milken Institute School of Public Health, The George Washington University, Washington, DC, 20052, USA; 4 Infectious Diseases Division, International Centre for Diarrhoeal Disease Research, ICDDR, B, Mohakhali, Dhaka, 1212, Bangladesh; 5 NHMRC Clinical Trials Centre, University of Sydney, Sydney, NSW, Australia; 6 Nutrition Research Division, International Centre for Diarrhoeal Disease Research, ICDDR, B, Dhaka, Bangladesh

**Keywords:** bovine lactoferrin, ferrous sulfate, iron-deficiency anemia, hemoglobin, ferritin, noninferiority trial, randomized controlled trial, women of reproductive age, Bangladesh, iron supplementation

## Abstract

**Background:**

Lactoferrin is an iron-binding glycoprotein present in secretions, including breast milk, with anti-inflammatory and antimicrobial properties. Previous studies suggest bovine lactoferrin (bLF) may improve hemoglobin and iron status; however, most evidence comes from high-income countries, and data from resource-limited settings remain limited.

**Objectives:**

This study aimed to determine whether oral bLF is noninferior to ferrous sulfate for improving hemoglobin among nonpregnant women with iron-deficiency anemia (IDA) in Bangladesh. We hypothesized that 200 mg or 400 mg bLF would be noninferior to 60 mg elemental iron as ferrous sulfate after 12 wk, using a prespecified noninferiority margin of −0.25 g/dL.

**Methods:**

We conducted a double-blind, randomized, parallel-group, noninferiority trial in Dhaka, Bangladesh. Women aged 18 to 49 y with IDA were randomized 1:1:1 to receive 60 mg elemental iron as ferrous sulfate, 200 mg bLF, or 400 mg bLF, each given daily with 400 μg folic acid for 12 wk. Ferrous sulfate was the active control. The primary outcome was hemoglobin change from baseline to 12 wk in the per-protocol population.

**Results:**

Of 3722 women screened, 555 were randomly assigned to receive 186 to 200 mg bLF, 185 to 400 mg bLF, and 184 to ferrous sulfate. Mean hemoglobin change at 12 wk was −0.2 ± 0.8 g/dL, 0.0 ± 0.9 g/dL, and 1.1 ± 1.3 g/dL, respectively. Compared with ferrous sulfate, mean differences were −1.2 g/dL (95% CI: −1.6 to −0.9) and −1.1 g/dL (95% CI: −1.4 to −0.8) for 200 mg and 400 mg bLF, respectively (*P* < 0.0001). Adverse events were comparable across groups.

**Conclusions:**

In nonpregnant Bangladeshi women aged 18 to 49 y with IDA, both bLF doses were inferior to ferrous sulfate for improving hemoglobin after 12 wk. These findings do not support bLF as a substitute for ferrous sulfate in similar settings.

This trial is registered with the Australian New Zealand Clinical Trials Registry as ACTRN12617001455358 (https://www.anzctr.org.au/Trial/Registration/TrialReview.aspx?id=373059).

## Introduction

Iron-deficiency anemia (IDA) poses a major global health challenge, contributing substantially to the burden of years lived with disability (∼33.3 million) [1,2]. In 2023, an estimated 30.5% of nonpregnant women and 35.5% of pregnant women worldwide were anemic, primarily due to iron deficiency [1,3]. In Bangladesh, the prevalence of anemia among all women was recorded at 41.8%, with 6.8% of women experiencing moderate to severe anemia [4]. A recent study in Bangladesh revealed an even higher prevalence of anemia among pregnant women, reaching 48.3% [5].

Daily oral iron supplementation is a widely recommended practice for preventing and treating IDA. WHO recommends daily supplementation with 30 to 60 mg elemental iron for 3 consecutive months for nonpregnant women of reproductive age in settings where the prevalence of anemia is >40% [6]. However, the effectiveness of preventive and therapeutic oral iron interventions is often limited by poor adherence, largely due to gastrointestinal side effects and inflammation-driven increases in hepcidin, which reduce intestinal iron absorption and iron release from body stores [7,8]. Gastrointestinal side effects can also be dose-limiting; an estimated 30% of patients experience side effects that deter adherence [[9], [10], [11], [12]].

Lactoferrin is a glycoprotein found in body secretions, including breast milk, which binds to iron and has anti-inflammatory and antioxidant properties [13,14]. Bovine lactoferrin (bLF), a dairy protein, is structurally similar to human lactoferrin. It can improve iron status through multiple mechanisms as follows: *1*) it binds to iron with high affinity and is absorbed through specific receptors on gut lining cells, separate from those for inorganic iron; *2*) its structure prevents degradation by stomach acid, allowing it to be taken orally and remain active; *3*) it suppresses inflammatory responses that may inhibit the utilization of iron. Notably, whereas a therapeutic dose of 200 to 400 mg of apo-lactoferrin contains only a fraction of the elemental iron found in 60 mg of ferrous sulfate, its efficacy is attributed to its high bioavailability and its ability to modulate systemic iron homeostasis rather than providing a large mineral load. Lactoferrin is also an effective inhibitor and microbicide for various microorganisms [13,14].

Several studies evaluated the effects of bLF supplementation in pregnant and nonpregnant women [13,[15], [16], [17]]. Most of these studies were conducted in high-income settings. A systematic review and meta-analysis compared the efficacy of oral lactoferrin to ferrous sulfate in treating IDA [18]. The review reported that lactoferrin improved hemoglobin compared with ferrous sulfate, with a weighted mean difference of 1.18 g/dL (*P* < 0.0001). One trial in 100 women that was included in the analysis was later retracted [16].

Given the promising outcomes observed in prior studies, we investigated bLF among women in Bangladesh, a low-resource setting with a high burden of IDA. Using a robust double-blind, randomized design with an adequate sample size, we aimed to evaluate whether bLF could serve as an effective alternative to ferrous sulfate for IDA among nonpregnant women of reproductive age. We hypothesized that doses of 200 mg and/or 400 mg of bLF would be noninferior to the universally recommended daily dose of 60 mg elemental iron as ferrous sulfate for treating IDA.

## Methods

### Study design and inclusion criteria

This was a parallel-group, 3-arm, double-blind, noninferiority, individually randomized controlled trial and a collaborative project between the University of Sydney, Australia, and International Centre for Diarrhoeal Disease Research, Bangladesh (icddr,b). The study was conducted in Mirpur, a suburb of Dhaka, Bangladesh, with a population of approximately 1 million. The study included nonpregnant, nonlactating women aged 18 to 49 y with IDA, defined as hemoglobin concentrations <12 g/dL and serum ferritin <30 μg/L [19], who were willing to attend scheduled follow-up visits. In addition to hemoglobin and ferritin, participants were also screened for diabetes mellitus and thalassemia trait. Participants were excluded from the study if they had severe anemia (hemoglobin <7 g/dL), had received a blood transfusion in the preceding months, had a history of tuberculosis, were currently taking other iron supplements, had thalassemia trait or diabetes mellitus, or had a known allergy or hypersensitivity to milk proteins or iron products. Those diagnosed with severe anemia, thalassemia trait, or diabetes mellitus during screening were also referred to a local health facility.

The study was approved by the ethical review committee of icddr,b and the human research ethics committee of the University of Sydney, and we also received regulatory clearance for the investigational product bLF from the Directorate General of Drug Administration, Bangladesh. All participants provided written informed consent. The consent form was provided in Bangla and read aloud to participants unable to read.

### Randomization and masking

In this study, eligible women were randomly assigned 1:1:1 to 1 of 3 treatment regimens using computer-generated numbers: WHO’s recommended regimen of daily supplementation with 60 mg elemental iron as ferrous sulfate and 400 μg folic acid; 200 mg bLF and 400 μg folic acid; and 400 mg bLF and 400 μg folic acid. The random allocation sequence was generated in Stata 18 (StataCorp LLC, College Station, TX, USA) by the trial statistician using unstratified block randomization (fixed block sizes of 9). Participants were assigned to these blocks in chronological order upon enrolment to ensure a continuous balance among the 3 study arms.

No study personnel involved in participant enrolment or assignment to interventions had access to the random allocation sequence. Allocation concealment was strictly maintained to ensure the integrity of the randomization process. The study physician prepared opaque, sequentially numbered, sealed envelopes and identically packaged treatment kits in accordance with the allocation sequence. At enrolment, the study physician opened the next sequential envelope and dispensed the corresponding prenumbered kit. To ensure the integrity of the study, throughout the trial, participants, site staff, investigators, data managers, and statisticians were masked to the treatment allocation.

### Procedures

Each treatment regimen comprised 3 uniformly shaped and colored capsules, as outlined in Table 1. Eskayef Bangladesh Limited, a leading pharmaceutical company in Bangladesh, produced all treatment capsules for this trial. The ferrous sulfate formulation was based on standard iron-folic acid supplementations regimen used in Bangladesh and internationally. All supplements were encapsulated in gelatin capsules.

**TABLE 1:** Composition of daily trial treatments by randomized group

Treatment group	Green capsule	Red capsule	White capsule
bLF 200 mg plus	200 mg bLF	Placebo	400 μg folic acid
bLF 400 mg plus	200 mg bLF	200 mg bLF	400 μg folic acid
Ferrous sulfate plus	60 mg elemental iron as ferrous sulfate	Placebo	400 μg folic acid

All participants were instructed to take one capsule from each color-coded blister pack once daily.

Abbreviations: bLF, bovine lactoferrin; bLF 200 mg plus, 200 mg bovine lactoferrin plus 400 μg folic acid; bLF 400 mg plus, 400 mg bovine lactoferrin plus 400 μg folic acid; ferrous sulfate plus, 60 mg elemental iron as ferrous sulfate plus 400 μg folic acid.

At enrolment, a trained public health physician provided standardized verbal instructions on supplement administration. Participants were advised to take their assigned supplement 30 min before meals. This timing was recommended to minimize the inhibitory effects of food on iron absorption. However, participants were advised that the supplement could be taken with a meal if gastrointestinal side effects occurred, to support tolerability and adherence. The prescribed oral treatment was continued for 12 wk.

All eligible women were contacted through household visits and apprised of the study details, including the number of clinic visits required for comprehensive hematological assessments. Upon obtaining informed consent, participants underwent screening for anemia by capillary blood sampling using a point-of-care HemoCue 201+ System. If hemoglobin concentrations were <12 g/dL, participants received a screening card. They were invited to visit the study clinic within 48 to 72 hours to undergo a serum ferritin test to screen for iron deficiency.

During the second screening phase, a qualified laboratory technician collected a venous blood sample, which was sent to the Immunobiology, Nutrition, and Toxicology Laboratory at icddr,b for evaluation of serum ferritin concentrations. On the same day, a rapid blood sugar test was performed to rule out diabetes mellitus. Participants with ferritin concentrations <30 μg/L were further assessed for thalassemia trait, following guidance from the Directorate General of Drug Administration research review committee in Bangladesh. Enrolment into the study was contingent upon meeting all eligibility criteria, and treatment initiation occurred within 7 d of the second screening.

Once enrolled, C-reactive protein (CRP), α-1-acid glycoprotein (AGP), and hepcidin were measured from the venous blood sample collected during the screening process. A baseline assessment was conducted soon after enrolment to collect sociodemographic information, reproductive history, current health status, anthropometry, and dietary intake. Data on dietary intake were collected using a validated 7-d semi-quantitative Food Frequency Questionnaire. Standardized food photographs from the National Nutrition Survey of Bangladesh 2011 were used by data collectors to determine serving amounts. Bangladesh’s Food Composition Table was used to estimate daily dietary iron intake [20,21]. First, cooked food weights were converted to raw food weights using appropriate conversion factors from the Food Composition Table. Daily iron intake per 100 g of raw food consumed was then estimated using the following formula: Iron consumption per day = (Per day intake × retention factor × nutrient value) / (100 × yield factor) [20,21].

Follow-up visits at the study clinic were scheduled at 4, 8, and 12 wk after the start of treatment. On each visit, every participant was seen by a study physician at the clinic. The study physician collected data on self-reported side effects, capsule intake, and care-seeking, defined as any consultation with a health care provider, clinic or hospital visit, medication use, or treatment sought for intercurrent illness, and provided the next month’s supply of capsules. Further measurements of serum hemoglobin, serum ferritin, CRP, and AGP were conducted at 4, 8, and 12 wk. Hepcidin was additionally measured at 12 wk.

Data from laboratory analyses were linked to the respective clinical data by a unique identifier. Safety was assessed by recording information on the type, duration, seriousness, and intensity of adverse events, as well as their relationship to the study drug and outcomes. Clinical information from each visit was documented in electronic data capture forms.

Hemoglobin was measured by the UV-1601 UV-VIS Spectrophotometer (SHIMADZU). Serum ferritin was measured using a Cobas e601 automated immunoassay analyzer (Roche Diagnostics), CRP and AGP were assessed using a Cobas c311 clinical chemistry analyzer (Roche Diagnostics). Hepcidin was measured by quantitative sandwich ELISA (Human Hepcidin Immunoassay, R&D Systems). Ferritin concentrations were adjusted for inflammation using Thurnham et al.’s [22] correction factors. This approach accounts for the artificial elevation of ferritin during subclinical inflammation by classifying participants into inflammation groups using CRP and AGP. Inflammation was classified as no inflammation (CRP ≤5 mg/L and AGP ≤1.0 g/L), incubation (CRP >5 mg/L and AGP ≤1.0 g/L), early convalescence (CRP >5 mg/L and AGP >1.0 g/L), or late convalescence (CRP ≤5 mg/L and AGP >1.0 g/L). Ferritin values in the inflammation groups were then adjusted using arithmetic correction factors derived from the meta-analysis by Thurnham et al. [22] to provide inflammation-adjusted ferritin concentrations and reduce the potential misclassification of iron deficiency.

### Outcomes

The primary outcome was the mean change in hemoglobin from baseline to week 12 to assess noninferiority of daily bLF 200 mg or 400 mg, or both, compared with 60 mg elemental iron as ferrous sulfate. Key secondary outcomes were changes in serum ferritin from baseline to week 12 with daily bLF 200 mg or 400 mg, or both, compared with 60 mg elemental iron as ferrous sulfate. Other secondary endpoints included changes in hepcidin; prevalence of anemia, iron deficiency, IDA, and the occurrence of adverse events across treatment groups.

Anemia was defined as a hemoglobin concentration <12 g/dL. Serum ferritin <30 μg/L was used to define iron deficiency because concurrent inflammation was assessed, and WHO guidance allows higher ferritin thresholds or inflammation adjustment when infection or inflammation is present. IDA was diagnosed if the hemoglobin concentration was <12 g/dL and the serum ferritin concentration was <30 μg/L [24,25]. We summarized the proportion of women with hepcidin <2.5 ng/mL as an exploratory biomarker of low hepcidin. Adverse events were defined as any unfavorable medical occurrence, including signs and symptoms associated with the research procedure or trial intervention, regardless of whether they were deemed related to the women’s participation in the research.

### Statistical analysis

The study was designed as a noninferiority trial to determine whether lactoferrin 200 mg, 400 mg, or both, was noninferior to 60 mg elemental iron as ferrous sulfate with respect to the primary endpoint of change in hemoglobin after 12 wk. We defined a noninferiority margin of −0.25 g/dL. This was based on evidence indicating that the mean effect of oral iron over placebo is ∼1.0 g/dL [26,27]. A margin of 0.25 g/dL preserves 75% of the active control’s effect, ensuring that any treatment effect of lactoferrin deemed noninferior would still represent a clinically meaningful improvement over no supplementation. This margin is consistent with the smallest clinically acceptable difference that would not alter the primary treatment decision for IDA in this population.

A sample size of 483 (i.e., 161 subjects per group) provided 80% power with a one-sided type I error of 0.025 (or equivalently with a 95% CI), to establish that 200 mg lactoferrin is noninferior to WHO’s recommended regimen of 60 mg elemental iron as ferrous sulfate, 400 mg lactoferrin is noninferior to WHO’s recommended regimen of 60 mg elemental iron as ferrous sulfate, and 200 mg lactoferrin is noninferior to 400 mg lactoferrin, assuming a hemoglobin SD of 0.8 g/dL in each group based on previous studies [26,27]. After factoring in dropout, the total sample size was adjusted to 555 subjects.

The primary analysis was conducted using a per-protocol approach, including only participants with available primary outcome data. In noninferiority trials, the per-protocol analysis is often preferred as it is more conservative and reduces the risk of falsely claiming noninferiority due to the diluting effects of noncompliance or dropouts [28].

The change in hemoglobin was calculated by subtracting the baseline hemoglobin concentration from the hemoglobin concentration measured after 12 wk. We used a *t*-test to calculate a 95% CI for the difference in mean change of hemoglobin between each lactoferrin dose and the ferrous sulfate group. If the lower limit of the CI was >−0.25 g/dL, we would conclude that the respective lactoferrin dosage was noninferior to 60 mg elemental iron as ferrous sulfate.

We also tested for superiority. The mean change in hemoglobin between the 2 lactoferrin doses and the ferrous sulfate group was compared using *t*-tests. We employed the Bonferroni correction to adjust for multiple comparisons when testing the superiority of each lactoferrin dose compared with the control group. We presented the frequency of adverse effects in all 3 groups.

We also performed an intention-to-treat (ITT) analysis as a sensitivity analysis. To handle missing data, we employed multiple imputation by chained equations. We used the mi impute chained procedure in Stata to simultaneously impute missing values for hemoglobin, ferritin, and hepcidin. The imputation model included baseline values; demographic covariates (screening age and maternal education level); and treatment assignment to preserve the randomization structure. We generated 100 imputed datasets. Once the missing values were imputed, the primary outcome was computed within each imputed set, and the results were then pooled for analysis. Finally, group-specific means and treatment effects were estimated using linear regression, with results pooled according to Rubin’s Rules to account for the uncertainty inherent in the imputation process.

Assumptions for *t*-tests and linear regression models were assessed by visual inspection of residual-compared with-fitted and normal quantile plots and by comparing group standard deviations. Categorical outcomes were compared using Pearson chi-square tests after confirming that expected cell counts were adequate. The checks did not reveal any significant departures that would change the interpretation of the primary or sensitivity analyses.

We used Stata software (version 18) for all statistical analyses. This trial is registered with the Australian New Zealand Clinical Trials Registry—ACTRN12617001455358 (https://www.anzctr.org.au/Trial/Registration/TrialReview.aspx?id=373059).

## Results

**FIGURE 1:**
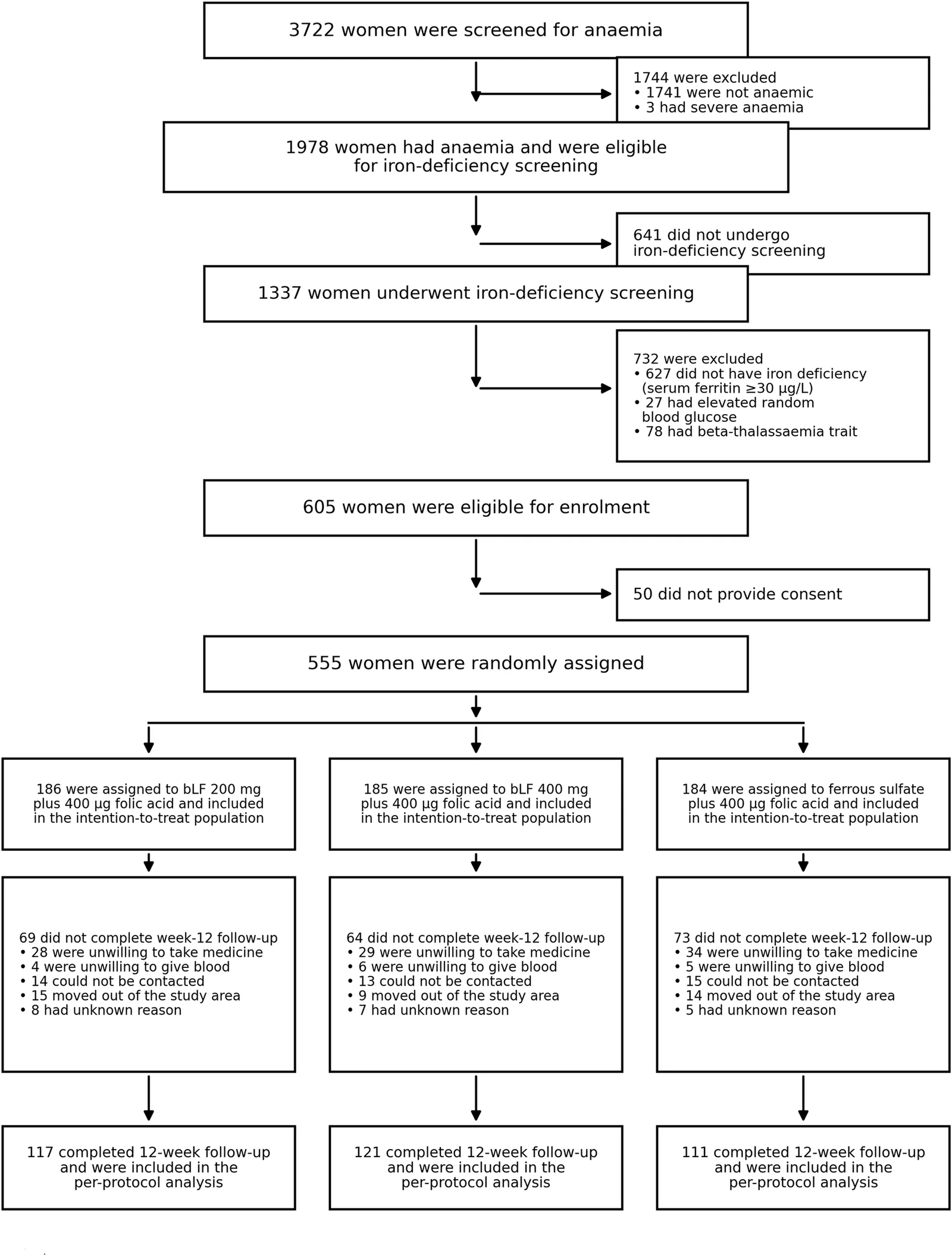
Trial profile. Participant flow from screening to randomization, follow-up, and inclusion in the primary per-protocol analysis. Women were screened for anemia using capillary hemoglobin measurement, followed by further screening for iron deficiency, diabetes mellitus and thalassaemia trait where indicated. Eligible women with iron-deficiency anemia were randomized in a 1:1:1 ratio to receive 200 mg bovine lactoferrin plus 400 μg folic acid, 400 mg bovine lactoferrin plus 400 μg folic acid, or 60 mg elemental iron as ferrous sulfate plus 400 μg folic acid daily for 12 wk. Reasons for exclusion and loss to follow-up are shown by treatment group. bLF, bovine lactoferrin.

Between 28 January, 2018, and 3 October, 2018, we screened 3722 women of reproductive age for anemia using capillary blood hemoglobin measurements (Figure 1). Of these, 1978 women (53.1%) were identified as anemic. Among them, 1337 women were further screened for iron deficiency; 627 were noniron deficient, 27 had elevated random blood sugar, 78 had β-thalassemia trait and 50 declined to participate further, leaving 555 participants enrolled in the trial. These participants were randomly assigned to 1 of 3 groups: ferrous sulfate (*n* = 184), 200 mg bLF (*n* = 186), or 400 mg bLF (*n* = 185). Of the 555 women enrolled, 349 (62.9%) completed the 12-wk follow-up and were included in the primary outcome analysis (*n* = 111 in the ferrous sulfate group, *n* = 117 in the 200 mg bLF group, and *n* = 121 in the 400 mg bLF group; Figure 1). Losses and exclusions after randomization for each study group are detailed in the study flow diagram (Figure 1).

The baseline characteristics of participants were generally balanced across the 3 treatment groups (Table 2). Maternal age, marital status, parity, education level, household size, and wealth distribution were comparable, with no notable differences likely to influence outcomes. Anthropometric measures, including weight, height, BMI, and mid-upper arm circumference, were also similar between groups. Biochemical markers—such as hemoglobin (measured by both automated analyzers and HemoCue), serum ferritin (unadjusted and inflammation adjusted), AGP, CRP, and hepcidin—showed comparable baseline levels across treatment arms. The prevalence of inflammation and its stages, as well as the proportion of women with hepcidin concentrations <2.5 ng/mL, were evenly distributed, supporting a well-randomized and balanced study cohort.

**TABLE 2:** Baseline demographic, anthropometric, and socioeconomic and biochemical characteristics of the randomized population

	bLF 200 mg plus (*n* = 186)	bLF 400 mg plus (*n* = 185)	Ferrous sulfate plus (*n* = 184)
Age, (y), mean ± SD	32.7 ± 8.7	31.4 ± 8.6	31.9 ± 8.7
Married (%)	88.7	87.0	85.3
Parity, mean ± SD	2.1 ± 1.7	2.0 ± 1.6	2.0 ± 1.7
Education			
No education, %	31.7	32.4	35.3
Primary incomplete, %	14.5	16.2	15.8
Primary complete, %	11.8	11.9	13.0
Secondary, %	28.5	29.2	26.1
Higher, %	13.4	10.3	9.8
Wealth index^1^			
Lowest, %	16.9	23.1	20.1
Second, %	21.4	20.8	17.8
Middle, %	20.8	18.5	20.7
Fourth, %	14.6	22.0	23.6
Highest, %	26.4	15.6	17.8
Household size, mean ± SD	4.4 ± 2.0	4.2 ± 1.7	4.3 ± 1.6
Women’s anthropometry			
Weight (kg), mean ± SD	54.5 ± 11.3	54.6 ± 11.0	55.4 ± 12.8
Height (cm), mean ± SD	150.4 ± 5.8	149.5 ± 6.1	150.2 ± 6.4
BMI (kg/m^2^), mean ± SD	24.1 ± 4.7	24.5 ± 5.0	24.5 ± 5.3
MUAC, mean ± SD	27.6 ± 3.9	27.8 ± 4.4	28.1 ± 4.0
Hemoglobin by automated analyzer, g/dL, mean ± SD	11.0 ± 1.3	11.2 ± 1.4	11.2 ± 1.3
Hemoglobin by HemoCue, g/dL, mean ± SD	10.5 ± 1.2	10.5 ± 1.2	10.6 ± 1.1
Serum ferritin, μg/L, mean ± SD	14.4 ± 8.0	14.6 ± 7.8	14.7 ± 7.7
Serum ferritin, inflammation adjusted, μg/L, mean ± SD^2^	13.5 ± 7.8	13.6 ± 7.5	13.8 ± 7.5
AGP (mg/dL), mean ± SD	71.5 ± 21.0	70.7 ± 21.3	75.4 ± 52.3
AGP >100 mg/dL (1.0 g/L), %	8.7	9.4	8.8
CRP (mg/L)	2.9 ± 3.8	2.8 ± 4.2	2.9 ± 3.8
CRP >5.0 mg/L, %	17.9	13.8	16.5
Inflammation (with stages)			
No inflammation	80.1	81.1	79.4
Incubation	10.2	7.6	10.9
Early convalescence	8.6	8.1	6.5
Late convalescence	1.1	3.2	3.3
Hepcidin (ng/mL), mean ± SD	4.0 ± 4.8	4.2 ± 5.3	4.3 ± 4.7
Hepcidin <2.5 ng/mL, %	55.9	51.9	50.0

Data are mean ± SD or percentages, as indicated.

Abbreviations: AGP, α-1-acid glycoprotein; bLF 200 mg plus, 200 mg bLF plus 400 μg folic acid; bLF 400 mg plus, 400 mg bLF plus 400 μg folic acid; CRP, C-reactive protein; ferrous sulfate plus, 60 mg elemental iron as ferrous sulfate plus 400 μg folic acid; MUAC, mid-upper arm circumference.

1 Ferritin concentrations were adjusted for inflammation using Thurnham et al.’s correction factors. Inflammation stages were defined as: no inflammation, CRP ≤5 mg/L and AGP ≤1.0 g/L; incubation, CRP >5 mg/L and AGP ≤1.0 g/L; early convalescence, CRP >5 mg/L and AGP >1.0 g/L; and late convalescence, CRP ≤5 mg/L and AGP >1.0 g/L.

2 The household wealth index was constructed using principal component analysis of household assets, drinking water source and sanitation. No statistically significant between-group differences were observed for the listed baseline variables.

Baseline demographics and clinical characteristics were also similar across all 3 groups in the per-protocol population (Supplementary Table S1). At baseline, over 98% of participants attended their assigned visit, with similar high compliance across the 3 groups. Follow-up visit attendance declined from 98.6% at baseline to 62.9% at week 12 and was similar across treatment groups (Supplementary Table S2); reasons for dropout were also similar by group (Supplementary Table S3).

**FIGURE 2:**
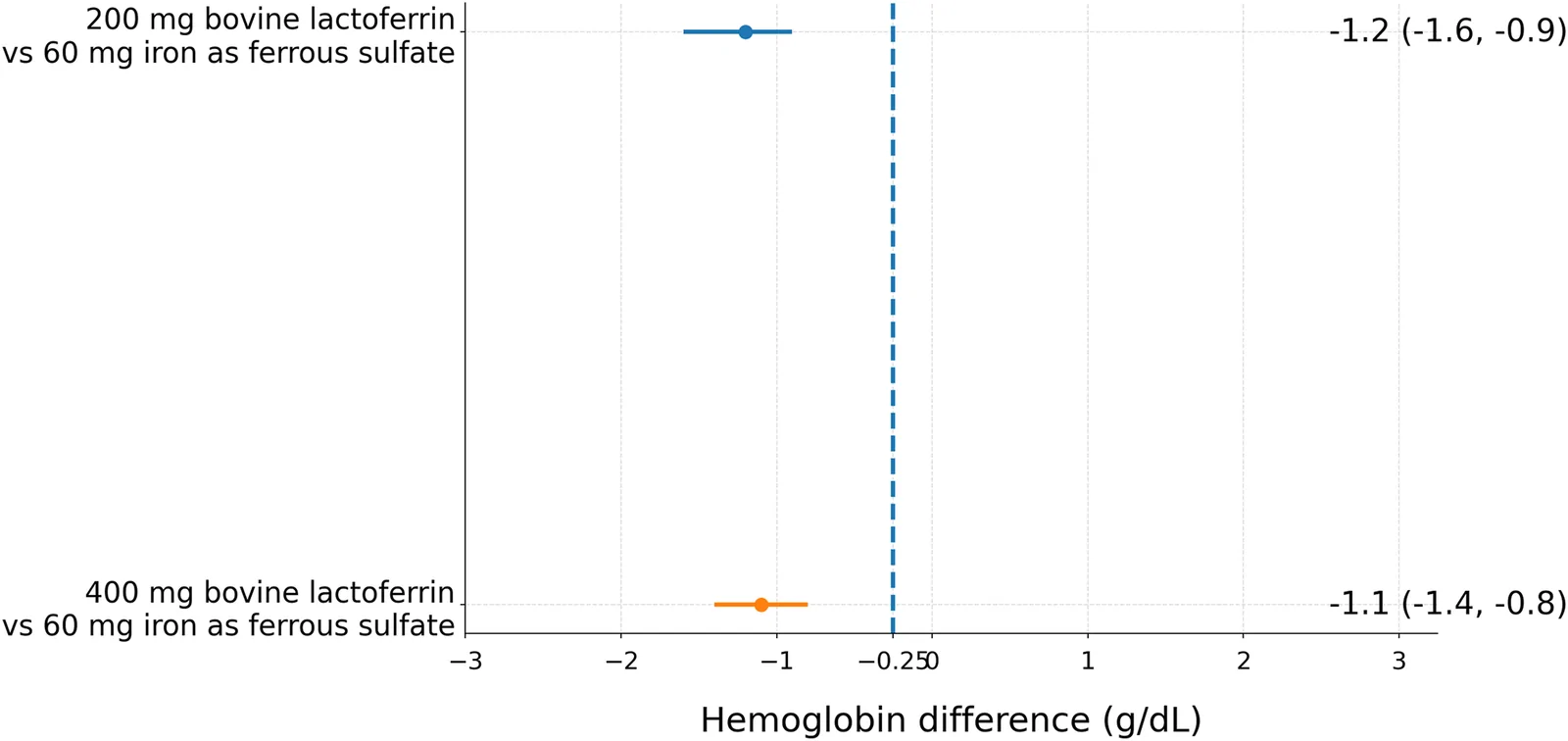
Noninferiority analysis of change in hemoglobin from baseline to week 12 in the per-protocol population. Values show the mean difference in hemoglobin change from baseline to week 12 for each bovine lactoferrin group compared with the ferrous sulfate group, with 95% CIs. Treatment differences are expressed as bovine lactoferrin minus ferrous sulfate. The vertical dotted line indicates the prespecified noninferiority margin of −0.25 g/dL. Noninferiority would be concluded if the lower limit of the 95% CI was >−0.25 g/dL. bLF, bovine lactoferrin; CI, confidence interval.

The mean change in hemoglobin at 12 wk was −0.2 ± 0.8 g/dL in the 200 mg bLF group and 0.0 ± 0.9 g/dL in the 400 mg group, compared with 1.1 ± 1.3 g/dL in the ferrous sulfate group (Table 3; Figure 2). The estimated treatment differences relative to ferrous sulfate were −1.2 g/dL (95% CI: −1.6 to −0.9) for 200 mg bLF and −1.1 g/dL (95% CI: −1.4 to −0.8) for 400 mg bLF. In both groups, the CIs were entirely below the prespecified noninferiority margin of −0.25 g/dL, indicating that bLF was inferior to ferrous sulfate for improving hemoglobin.

**TABLE 3:** Primary and secondary continuous outcomes at week 12 in the per-protocol population.

	bLF 200 mg plus (*n* = 117)	bLF 400 mg plus (*n* = 121)	Ferrous sulfate plus (*n* = 111)
Hemoglobin			
Baseline, g/dL, mean ± SD	10.9 ± 1.3	11.2 ± 1.4	11.2 ± 1.2
Week 12, g/dL, mean ± SD	10.7± 1.3	11.2 ± 1.4	12.3 ± 0.8
Change from baseline at week 12^1^	−0.2 ± 0.8	0.0 ± 0.9	1.1 ± 1.3
Treatment difference vs FS (95% CI)^2^	−1.2 (−1.6, −0.9)	−1.1 (−1.4, −0.8)	—
*P* value^3^	<0.0001	<0.0001	
Ferritin			
Baseline μg/L, mean ± SD	14.2 ± 8.1	14.7 ± 7.6	14.4 ± 7.4
Week 12, μg/L, mean ± SD	17.4 ± 13.4	18.1 ± 17.3	55.8 ± 35.7
Change from baseline at week 12^1^	3.2 ± 10.8	3.5 ± 15.1	41.4 ± 34.7
Treatment difference vs FS (95% CI)^2^	−38.2 (−45.4, −31.1)	−37.9 (−45.0, −30.8)	—
*P* value^3^	<0.0001	<0.0001	
Ferritin, inflammation adjusted (μg/L)			
Baseline, μg/L, mean ± SD	13.5 ± 7.8	13.6 ± 7.5	13.8 ± 7.5
Week 12, μg/L, mean ± SD	15.6 ± 11.9	16.8 ± 17.1	50.8 ± 31.8
Change from baseline at week 12^1^	2.6 ± 9.6	3.0 ± 14.9	37.6 ± 31.0
Treatment difference vs FS (95% CI)^2^	−35.0 (−41.5, −28.5)	−34.7 (−41.1, −28.2)	—
*P* value^3^	<0.0001	<0.0001	
Hepcidin			
Baseline, ng/mL, mean ± SD	3.7 ± 4.5	4.3 ± 5.7	3.9 ± 3.7
Week 12, ng/mL, mean ± SD	4.7 ± 5.9	4.9± 6.3	19.7 ± 16.8
Change from baseline at week 12^1^	1.0 ± 5.6	0.5 ± 6.7	15.9 ± 16.8
Treatment difference vs FS (95% CI)^2^	−14.9 (−18.3, −11.4)	−15.4 (−18.8, −11.9)	—
*P* value^3^	<0.0001	<0.0001	

Data are mean ± SD unless otherwise stated.

Abbreviations: bLF, bovine lactoferrin; bLF 200 mg plus, 200 mg bovine lactoferrin plus 400 μg folic acid; bLF 400 mg plus, 400 mg bovine lactoferrin plus 400 μg folic acid; CI, confidence interval; FS, ferrous sulfate; ferrous sulfate plus, 60 mg elemental iron as ferrous sulfate plus 400 μg folic acid.

1 Change is week 12 minus baseline.

2 Treatment differences are bLF minus ferrous sulfate.

3 *P* values are from *t*-tests comparing each bLF group with ferrous sulfate, with Bonferroni correction where applicable.

The mean ferritin concentration increased substantially in the ferrous sulfate group after 12 wk, from 14.4 ± 7.4 μg/L to 55.8 ± 35.7 μg/L, compared with small increases in the bLF groups. The mean changes were 3.2 ± 10.8 μg/L in the 200 mg bLF group, 3.5 ± 15.1 μg/L in the 400 mg bLF group, and 41.4 ± 34.7 μg/L in the ferrous sulfate group. Compared with ferrous sulfate, the estimated treatment differences were −38.2 μg/L (95% CI: −45.4 to −31.1) for 200 mg bLF and −37.9 μg/L (95% CI: −45.0 to −30.8) for 400 mg bLF (Table 3). Mean hemoglobin and ferritin concentrations over follow-up are shown in Supplementary Figure S1.

Hepcidin concentrations increased in all treatment groups. However, the increase was minimal in the bLF groups, with a mean increase of 1.0 ± 5.6 ng/mL in the 200 mg bLF group and 0.5 ± 6.7 ng/mL in the 400 mg bLF group. In contrast, the ferrous sulfate group exhibited a substantial 15.9 ± 16.8 ng/mL increase. Compared with ferrous sulfate, the estimated treatment differences were −14.9 ng/mL (95% CI: −18.3 to −11.4) for 200 mg bLF and −15.4 ng/mL (95% CI: −18.8 to −11.9) for 400 mg bLF (Table 3).

Inflammatory-marker concentrations did not differ meaningfully between treatment groups over the 12-wk intervention. CRP increased by 0.67 mg/L in the bLF 200 mg group, 0.29 mg/L in the bLF 400 mg group, and 0.44 mg/L in the ferrous sulfate group, with no significant between-group difference. AGP changed by −0.09 mg/dL, 2.27 mg/dL, and −6.38 mg/dL in the bLF 200 mg, bLF 400 mg, and ferrous sulfate groups, respectively, again with no significant between-group difference. Detailed CRP and AGP changes from baseline to week 12 are shown in Table 4.

**TABLE 4:** Changes in inflammatory markers from baseline to week 12 in the per-protocol population.

	bLF 200 mg plus (*n* = 117)	bLF 400 mg plus (*n* = 121)	Ferrous sulfate plus (*n* = 111)
CRP			
Baseline, (mg/L), mean ± SD	2.92 ± 3.49	2.49 ± 3.16	3.24 ± 4.36
Week 12, (mg/L), mean ± SD	3.60 ± 4.68	2.79 ± 3.11	3.74 ± 3.77
Change from baseline at week 12^1^	0.67 ± 2.82	0.29 ± 2.79	0.44 ± 4.13
Treatment difference vs FS (95% CI)^2^	0.23 (−0.83, 1.28)	−0.16 (−1.20, 0.89)	Reference
*P* value^3^	1.000	1.000	
AGP			
Baseline, (mg/dL), mean ± SD	71.68 ± 20.07	70.31 ± 20.78	78.65 ± 64.90
Week 12, (mg/dL), mean ± SD	71.57 ± 21.45	72.74 ± 20.87	72.32 ± 18.02
Change from baseline at week 12^1^	−0.09 ± 16.86	2.27 ± 18.69	−6.38 ± 64.83
Treatment difference vs FS(95% CI)^2^	6.29 (−6.33, 18.90)	8.65 (−3.86, 21.16)	Reference
*P* value^3^	0.694	0.292	

Data are mean ± SD unless otherwise stated. Change is week 12 minus baseline. Treatment differences are bLF minus ferrous sulfate. *P* values compare each bLF group with ferrous sulfate.

Abbreviations: AGP, α-1-acid glycoprotein; bLF, bovine lactoferrin; bLF 200 mg plus, 200 mg bovine lactoferrin plus 400 μg folic acid; bLF 400 mg plus, 400 mg bovine lactoferrin plus 400 μg folic acid; CI, confidence interval; CRP, C-reactive protein; ferrous sulfate plus, 60 mg elemental iron as ferrous sulfate plus 400 μg folic acid.

The sensitivity analysis using the ITT population with multiple imputation yielded results consistent with the primary per-protocol analysis (Table 5). In the ITT population, the mean change in hemoglobin was −0.24 g/dL in the 200 mg bLF group and 0.09 g/dL in the 400 mg bLF group, compared with a gain of 1.00 g/dL in the ferrous sulfate group. The treatment differences relative to ferrous sulfate remained significant for both the 200 mg group (−1.24 g/dL; 95% CI: −1.50, −0.99; *P* < 0.0001) and the 400 mg group (−0.91 g/dL; 95% CI: −1.17, −0.65; *P* < 0.0001). Consistent with the primary analysis, the ferrous sulfate group showed substantially higher increases in both ferritin (38.27 ± 2.65 μg/L) and hepcidin (14.35 ± 1.33 ng/mL) than the bLF groups. In the ITT analysis, the treatment differences for ferritin relative to ferrous sulfate were −37.61 (−43.89, −31.33) μg/L for the 200 mg group and −30.69 (−36.40, −24.97) μg/L for the 400 mg group (*P* < 0.0001). Overall, the ITT analysis confirmed the primary finding: both doses of bLF were inferior to ferrous sulfate in improving iron status and hemoglobin concentrations over 12 wk.

**TABLE 5:** Primary and secondary continuous outcomes in the intention-to-treat population after multiple imputation.

	bLF 200 mg plus (*n* = 186)	bLF 400 mg plus (*n* = 185)	ferrous sulfate plus (*n* = 184)
Hemoglobin			
Baseline, g/dL, mean ± SE	10.98 ± 0.09	11.17 ± 0.10	11.22 ± 0.10
Week 12, g/dL, mean ± SE	10.74 ± 0.10	11.26 ± 0.11	12.22 ± 0.10
Change from baseline at week 12^1^	−0.24 ± 0.08	0.09 ± 0.08	1.00 ± 0.11
Treatment difference vs FS (95% CI)^2^	−1.24 (−1.50, −0.99)	−0.91 (−1.17, −0.65)	—
*P* value^3^	<0.0001	<0.0001	
Ferritin			
Baseline μg/L, mean ± SE	14.40 ± 0.59	14.63 ± 0.57	14.71 ± 0.57
At week 12 μg/L, mean ± SE	15.06 ± 2.03	22.21 ± 1.90	52.98 ± 2.71
Change from baseline at week 12^1^	0.66 ± 1.95	7.58 ± 1.79	38.27 ± 2.65
Treatment difference vs FS (95% CI)^2^	−37.61 (−43.89, −31.33)	−30.69 (−36.40, −24.97)	—
*P* value^3^	<0.0001	<0.0001	
Ferritin, inflammation adjusted (μg/L)			
Baseline μg/L, mean ± SE	14.05 ± 0.57	14.16 ± 0.56	14.35 ± 0.56
At week 12 μg/L, mean ± SE	14.52 ± 1.94	21.48 ± 1.81	50.99 ± 2.57
Change from baseline at week 12^1^	0.54 ± 1.87	7.12 ± 1.73	36.71 ± 2.53
Treatment difference vs FS (95% CI)^2^	−36.17 (−42.18, −30.15)	−29.59 (−35.05, −24.13)	—
*P* value^3^	<0.0001	<0.0001	
Hepcidin			
Baseline ng/mL, mean ± SE	4.04 ± 0.35	4.20 ± 0.39	4.31 ± 0.35
At week 12 ng/mL, mean ± SE	3.80 ± 0.90	6.54 ± 0.82	18.65 ± 1.31
Change from baseline at week 12^1^	−0.23 ± 0.90	2.28 ± 0.83	14.35 ± 1.33
Treatment difference vs FS (95% CI)^2^	−14.58 (−17.56, −11.60)	−12.07 (−14.94, −9.20)	—
*P* value^3^	<0.0001	<0.0001	

NOTE. Data are mean ± SE unless otherwise stated. Missing values were imputed using multiple imputation by chained equations.

Abbreviations: bLF 200 mg plus, 200 mg bovine lactoferrin plus 400 μg folic acid; bLF 400 mg plus, 400 mg bovine lactoferrin plus 400 μg folic acid; CI, confidence interval; FS, ferrous sulfate; ferrous sulfate plus, 60 mg elemental iron as ferrous sulfate plus 400 μg folic acid; ITT, intention-to-treat.

1 Change is week 12 minus baseline.

2 Treatment differences are bLF minus ferrous sulfate.

3 P values are from pooled linear regression models after multiple imputation.

The prevalence of anemia declined in the reference group, from 72.0% at baseline to 36.9% at week 12, representing a reduction of 35.1 percentage points. In contrast, anemia prevalence increased slightly in both bLF groups, rising from 80.3% to 86.3% in the 200 mg group and from 67.7% to 71.9% in the 400 mg bLF group. Compared with ferrous sulfate, the differences in change in anemia prevalence at week 12 were 41.1 percentage points (95% CI: 25.5–56.7; *P* < 0.0001) for the 200 mg bLF group and 39.3 percentage points (95% CI: 23.8–54.7; *P* < 0.0001) for the 400 mg bLF group (Table 6). Baseline anemia was below 100% because eligibility was determined using screening capillary HemoCue hemoglobin, whereas baseline values were measured using venous automated analyzer samples. Previous evidence shows poor agreement between capillary and venous hemoglobin measurements [29].

**TABLE 6:** Secondary categorical outcomes in the per-protocol population.

	bLF 200 mg plus (*n* = 117)	bLF 400 mg plus (*n* = 121)	Ferrous sulfate plus (*n* = 111)
Anemia			
Baseline, % (95% CI)	80.3 (73.0, 87.7)	67.7 (59.3, 76.2)	72.0 (63.6, 80.5)
Week 12, % (95% CI)	86.3 (80.0, 92.6)	71.9 (63.8, 80.0)	36.9 (27.8, 46.1)
Change from baseline at week 12^1^	6.0 (−1.7, 13.7)	4.2 (−3.4, 11.6)	−35.1 (−46.7, −23.6)
Treatment difference vs FS(95% CI)^2^	41.1 (25.5, 56.7)	39.3 (23.8, 54.7)	—
*P* value^3^	<0.0001	<0.0001	
Low hepcidin at week 12 (hepcidin <2.5 ng/mL)			
Baseline, % (95% CI)	58.1 (49.0, 67.2)	50.4 (41.4, 59.5)	49.5 (40.1, 59.0)
Week 12, % (95% CI)	55.6 (46.4, 64.7)	52.9 (43.9, 61.9)	9.9 (4.3, 15.6)
Change from baseline at week 12^1^	−2.6 (−10.0, 4.8)	2.5 (−6.9, 11.9)	−39.6 (−49.6, −29.7)
Treatment difference vs FS (95% CI)^2^	37.1 (21.5, 52.6)	42.1 (26.7, 57.5)	—
*P* value^3^	<0.0001	<0.0001	
Iron deficiency			
Baseline, % (95% CI)	100	100	100
Week 12, % (95% CI)	85.5 (79.0, 92.0)	86.0 (79.7, 92.2)	21.6 (13.8, 29.4)
Change from baseline at week 12^1^	−14.5 (−21.0, −8.0)	−14.0 (−20.3, −7.8)	−78.4 (−86.2, −70.6)
Treatment difference vs FS (95% CI)^2^	63.8 (52.0, 75.7)	64.3 (52.6, 76.1)	—
*P* value^3^	<0.0001	<0.0001	
Iron-deficiency anemia			
Baseline, % (95% CI)	80.3 (73.0, 87.7)	67.8 (59.3, 76.2)	67.8 (59.3, 76.2)
Week 12, % (95% CI)	76.9 (69.2, 84.7)	64.5 (55.8, 73.1)	11.7 (5.6, 17.8)
Change from baseline at week 12^1^	−3.4 (−12.1, 5.2)	−3.3 (−11.3, 4.7)	−60.4 (−69.9, −50.8)
Treatment difference vs FS (95% CI)^2^	56.9 (41.8, 72.1)	57.1 (42.0, 72.1)	—
*P* value^3^	<0.0001	<0.0001	

Data are percentages with 95% CIs unless otherwise stated. Low hepcidin is reported as an exploratory biomarker and should not be interpreted as a validated clinical readiness index.

Abbreviations: bLF 200 mg plus, 200 mg bovine lactoferrin plus 400 μg folic acid; bLF 400 mg plus, 400 mg bovine lactoferrin plus 400 μg folic acid; CI, confidence interval; FS, ferrous sulfate; ferrous sulfate plus, 60 mg elemental iron as ferrous sulfate plus 400 μg folic acid.

1 Change is week 12 minus baseline in percentage points.

2 Treatment differences are bLF minus ferrous sulfate in percentage points.

3 *P* values compare each bLF group with ferrous sulfate.

The prevalence of low hepcidin (<2.5 ng/mL) declined substantially in the ferrous sulfate group, from 49.5% at baseline to 9.9% at week 12. In contrast, prevalence remained relatively stable in the bLF groups, decreasing slightly from 58.1% to 55.6% in the 200 mg group and increasing from 50.4% to 52.9% in the 400 mg group. This low-hepcidin measure is exploratory and should not be interpreted as a validated clinical readiness index.

The prevalence of iron deficiency declined markedly in the reference group (ferrous sulfate), from 100% at baseline to 21.6% at week 12. In comparison, the reduction was modest in both bLF groups: from 100% to 85.5% in the 200 mg group and to 86.0% in the 400 mg group. At week 12, the prevalence of iron deficiency remained substantially higher in both bLF groups than in the ferrous sulfate group, with treatment differences of 63.8 percentage points (95% CI: 52.0–75.7; *P* < 0.0001) and 64.3 percentage points (95% CI: 52.6–76.1; *P* < 0.0001), respectively (Table 6).

The prevalence of IDA declined substantially in the reference group (ferrous sulfate), from 67.8% at baseline to 11.7% at week 12. In contrast, prevalence remained largely unchanged in the bLF groups: decreasing slightly from 80.3% to 76.9% in the 200 mg group, and from 67.8% to 64.5% in the 400 mg group. At week 12, the prevalence of IDA was significantly lower in the ferrous sulfate group than in either lactoferrin group, with treatment differences of 56.9 percentage points (95% CI: 41.8–72.1; *P* < 0.0001) for the 200 mg bLF group and 57.1 percentage points (95% CI: 42.0–72.1; *P* < 0.0001) for the 400 mg bLF group (Table 6). Logistic regression models adjusted for baseline status showed consistent findings for endline anemia, iron deficiency, and IDA (Supplementary Table S4).

The frequency of adverse events was generally comparable across treatment groups, except for constipation and black stool, which were significantly more common in the ferrous sulfate group (Table 7). Black stool was reported in 10.4% of participants in the ferrous sulfate group at day 30 and in 9.0% at day 60, whereas it was nearly absent in the bLF groups. Similarly, constipation was more frequent in the ferrous sulfate group at day 30 (9%) than in the bLF 200 mg (2.1%) and 400 mg (1.4%) groups.

**TABLE 7:** Safety outcomes for the per-protocol population.

	After 30 d				After 60 d			
	bLF 200 mg plus (*n* = 142)	bLF 400 mg plus (*n* = 142)	Ferrous sulfate plus (*n* = 134)	*P* value^1^	bLF 200 mg plus (*n* = 124)	bLF 400 mg plus (*n* = 126)	Ferrous sulfate plus (*n* = 122)	*P* value^1^
Gastric irritation	14 (9.9)	11 (7.7)	14 (10.4)	0.717	15 (12.1)	11(8.8)	16 (13.1)	0.675
Vomiting	1 (0.7)	2 (1.4)	3 (2.2)	0.563	2 (1.6)	0 (0.0)	7 (5.7)	0.036
Nausea	0 (0.0)	1 (0.7)	1 (0.7)	0.596	6 (4.8)	0 (0.0)	6 (4.9)	0.118
Constipation	3 (2.1)	2 (1.4)	12 (9)	0.002	4 (3.2)	3 (2.4)	6 (4.9)	0.683
Heartburn	9 (6.3)	4 (2.8)	11 (8.2)	0.146	2 (1.6)	6 (4.8)	11 (9.0)	0.088
Diarrhea	3 (2.1)	6 (4.2)	2 (1.5)	0.327	1 (0.8)	1 (0.8)	8 (6.6)	0.021
Black stool	1 (0.7)	0 (0.0)	14 (10.4)	0.000	1 (0.8)	0 (0.0)	11 (9.0)	0.000

Data are *n* (%). Percentages are based on available participants at each assessment.

Abbreviations: bLF 200 mg plus, 200 mg bovine lactoferrin plus 400 μg folic acid; bLF 400 mg plus, 400 mg bovine lactoferrin plus 400 μg folic acid; ferrous sulfate plus, 60 mg elemental iron as ferrous sulfate plus 400 μg folic acid.

1 *P* values are from Pearson chi-square tests comparing proportions across the 3 treatment groups at each time point. Bonferroni correction was used for multiple corrections.

Although gastric irritation was the most commonly reported side effect, its incidence was similar across all groups. Other gastrointestinal symptoms, such as vomiting, nausea, heartburn, and diarrhea, occurred at low frequencies, with no consistent differences between groups, except for higher vomiting and diarrhea rates at day 60 in the ferrous sulfate group. We also estimated daily dietary iron intake from food consumption. Mean dietary iron intake ranged from 6.9 to 7.3 mg/day across the 3 groups and was comparable; >95% of participants had low dietary iron intake (<15 mg/day; Supplementary Table S5).

## Discussion

Our study represents the largest double-blind, randomized controlled trial to date evaluating oral bLF for the treatment of IDA in women. A total of 555 women with IDA were enrolled and randomly assigned to receive either 200 mg or 400 mg of bLF or the standard treatment of 60 mg elemental iron as ferrous sulfate. After 12 wk of supplementation, neither dose of bLF produced clinically meaningful improvements in serum hemoglobin or ferritin concentrations. These findings demonstrate a clear lack of efficacy for bLF in this population. In contrast, ferrous sulfate led to significant improvements in iron status, with mean hemoglobin differences favoring ferrous sulfate by 1.2 g/dL and 1.1 g/dL compared with the 200 mg and 400 mg bLF groups, differences were found favoring ferrous sulfate over both doses of lactoferrin (–38.2 and –37.9 μg/L, respectively).

Our findings contrast sharply with those of a prior systematic review and meta-analysis of oral lactoferrin trials, which concluded that bLF improved hemoglobin and ferritin concentrations more than ferrous sulfate did [18,30]. This meta-analysis, based on 8 trials (*n* = 1013), reported a weighted mean difference in hemoglobin of 1.18 g/dL in favor of lactoferrin. However, several limitations of the previous meta-analysis warrant consideration. First, most of the included trials were small-scale, increasing the risk of type I error (false-positive results). Second, 3 trials lacked proper randomization or blinding procedures, introducing potential bias that could compromise the validity of their findings; these concerns are summarized in the risk-of-bias assessment (Supplementary Figure S2). The article reporting the Egyptian trial was retracted in 2023, raising concerns regarding its reliability [16].

Several factors may explain why bLF was less effective in our Bangladeshi study population than in previous trials that reported improvements in hemoglobin and ferritin among women with IDA. An important consideration is whether sufficient dietary iron was available for lactoferrin-mediated effects to occur. Mean dietary iron intake in our study was only 6.9 to 7.3 mg/day across the 3 treatment groups, and >95% of participants had low dietary iron intake (<15 mg/day; Supplementary Table S5). Because bLF does not act as a conventional iron supplement, any potential benefit would depend largely on its ability to facilitate the absorption, transport, or utilization of iron already available from dietary or supplemental sources [[31], [32], [33]]. This is particularly relevant in diets where much of the available iron is nonheme iron, since nonheme iron absorption is strongly influenced by meal composition and can be inhibited by dietary phytates and polyphenols [34]. In a population with limited dietary iron substrate, lactoferrin-mediated effects would therefore be unlikely to translate into measurable improvements in ferritin or hemoglobin.

This interpretation is supported by the very low elemental iron contribution of the bLF product used in this trial. The product was unsaturated apo-lactoferrin, and, based on laboratory analysis showing an iron concentration of 127.99 mg/kg, the 200 mg and 400 mg daily doses supplied only ∼0.026 mg and 0.051 mg elemental iron, respectively. Several earlier studies used partially iron-saturated bLF, commonly described as 20% to 30% iron saturated; however, even these preparations supplied only very small amounts of elemental iron, ∼70 to 84 μg/day in total when 100 mg was administered twice daily [15]. Therefore, any clinical benefit observed in earlier studies is unlikely to be explained solely by direct iron delivery and may instead depend on sufficient dietary or supplemental iron, differences in iron bioavailability, and the biological effects of lactoferrin on iron transport and inflammatory regulation [[31], [32], [33], [34]]. Iron saturation may also influence lactoferrin stability, with holo-lactoferrin showing greater resistance to gastric digestion than apo-lactoferrin in adult studies [35].

The inflammatory-marker findings also require cautious interpretation. Changes in CRP and AGP from baseline to week 12 were small and comparable across treatment groups. The findings provide no clear evidence that bLF produced a greater systemic anti-inflammatory effect than ferrous sulfate. This interpretation is consistent with the hepcidin findings: both bLF groups showed substantially smaller hepcidin increases than the ferrous sulfate group, suggesting less activation of iron-regulatory feedback. However, this lower hepcidin response did not overcome the limited dietary iron supply or translate into improved ferritin or hemoglobin. Taken together, these findings suggest that low dietary iron availability, rather than hepcidin-mediated inflammatory blockade alone, likely contributed to the absence of a clinically meaningful effect of lactoferrin.

These findings suggest a potential role for bLF as an adjunct therapy rather than a stand-alone therapy. The co-administration of bLF with low-dose elemental iron may enhance iron absorption efficiency while attenuating hepcidin induction, thereby balancing efficacy and tolerability. This approach may be particularly relevant for populations prone to gastrointestinal side effects or poor adherence to conventional iron therapy. Future trials should formally evaluate combination regimens incorporating bLF with reduced iron doses, with hepcidin dynamics and fractional iron absorption as key mechanistic outcomes.

Although this study was conducted in an urban Bangladeshi setting, the findings are likely generalizable to many other low- and middle-income countries where IDA occurs in the context of low dietary iron intake, phytate-rich staple diets, chronic inflammation, and impaired gut function related to environmental enteric dysfunction. These shared contextual factors may limit the effectiveness of lactoferrin-based interventions that rely on sufficient dietary iron and intact absorptive capacity. Therefore, our findings suggest that bLF may not be a “one-size-fits-all” solution for IDA in resource-limited settings. We acknowledge the relatively high loss to follow-up as a limitation of this study. Although attrition can introduce selection bias, sensitivity analyses confirmed that baseline characteristics remained balanced across treatment groups and that the trial retained adequate power to detect the prespecified effects. Moreover, the magnitude and consistency of the observed differences favoring ferrous sulfate across the primary and key secondary outcomes make it unlikely that attrition alone explains the lack of efficacy observed with lactoferrin. Nevertheless, additional multicenter studies in diverse populations, with strategies to maximize retention, would further strengthen the evidence base before broader conclusions regarding the applicability of bLF across settings are drawn.

In conclusion, our study does not support the routine use of unsaturated bLF to improve iron status in nonpregnant women with IDA. Although lactoferrin may play a role in specific contexts, it appears ineffective in populations with low dietary iron intake. Future research should explore combined strategies, such as lactoferrin with low-dose iron or its use in populations with higher baseline iron availability, to better define its potential therapeutic role.

## Author contributions

The authors’ responsibilities were as follows : TMH contributed to conceptualization, study design, data analysis, data interpretation, funding acquisition, supervision, writing – original draft preparation, writing – review and editing; SI contributed to data collection, investigation, project administration, review and editing; NBA contributed to data collection, investigation, project administration, review and editing; SETB contributed to data collection, investigation, project administration; QSR contributed to data analysis; RR contributed to investigation, data collection, writing – review and editing; ATP contributed to data analysis, data interpretation, writing – review and editing; WTM contributed to data interpretation, writing – review and editing; SEA contributed to conceptualization, study design, supervision, writing – review and editing; MJD contributed to conceptualization, study design, funding acquisition, writing – review and editing; and all authors: read and approved the final manuscript.

## Data availability

De-identified analytical datasets will be made available upon request to the Institutional Review Board Coordinator at icddr,b via email at info@icddrb.org. Data sharing is contingent upon submission and approval of a formal proposal, which will undergo scientific review by the Institutional Review Board at icddr,b. If approved, data will be shared through a secure online platform in accordance with icddr,b’s published data access policies. Requestors will also be required to sign icddr,b’s standard Data Access Agreement before data release.

## Funding

Saving Lives at Birth and the UK Medical Research Council. The funders of the study had no role in study design, data collection, data analysis, data interpretation, or writing of the report. All authors had full access to all the data in the study and had final responsibility for the decision to submit for publication.

## Declaration of generative AI and AI-assisted technologies in the writing process

During the preparation of this work, the author(s) used ChatGPT Version 5.4 (OpenAI) to improve the clarity of some author-written paragraphs in the manuscript. After using this tool, the author(s) reviewed and edited the content as needed and take full responsibility for the content of the publication.

## Conflict of interest

The authors have no financial, personal, academic, or other relationships that could be perceived to influence the work reported in this manuscript.
