# Supplementary material for: Bovine Lactoferrin Compared With Ferrous sulfate for Treating Iron-Deficiency Anemia in Bangladeshi Women—A Randomized Controlled Trial
Source: J Nutr. 2026 Jun 17;156(8):101672. doi: 10.1016/j.tjnut.2026.101672 (PMC13494163; doi:10.1016/j.tjnut.2026.101672)
Supplement: Supplementary file 1 [file mmc1.docx]

**Bovine Lactoferrin compared to ferrous sulfate for treating Iron Deficiency Anemia in Bangladeshi women - a randomized controlled trial**

Tanvir M. Huda

**Supplemental Table S1: Baseline demographic, anthropometric, socioeconomic, and biochemical characteristics of the per-protocol population.**

| **Characteristic** | **bLF 200 mg plus (n=117)** | **bLF 400 mg plus (n=121)** | **Ferrous sulphate plus (n=111)** |
| --- | --- | --- | --- |
| Age, years, mean ± SD | 33.3 ± 8.5 | 31.4 ± 8.8 | 32.1 ± 9.0 |
| Married, % | 87.2 | 85.1 | 84.7 |
| Parity, mean ± SD | 2.1 ± 1.6 | 2.0 ± 1.5 | 2.2 ± 1.8 |
| **Education, %** | | | |
| No education | 23.1 | 30.6 | 29.7 |
| Primary incomplete | 15.4 | 17.4 | 17.1 |
| Primary complete | 13.7 | 12.4 | 9.9 |
| Secondary | 32.5 | 28.9 | 30.6 |
| Higher | 15.4 | 10.7 | 12.6 |
| **Wealth index, %** | | | |
| Lowest | 12.0 | 23.3 | 9.9 |
| Second | 23.1 | 21.7 | 18.0 |
| Middle | 20.5 | 18.3 | 22.5 |
| Fourth | 13.7 | 22.5 | 27.9 |
| Highest | 30.8 | 14.2 | 21.6 |
| Household size, mean ± SD | 4.5 ± 2.1 | 4.4 ± 1.8 | 4.5 ± 1.7 |
| **Women's anthropometry** | | | |
| Weight (kg), mean ± SD | 55.7 ± 11.8 | 54.7 ± 11.1 | 56.7 ± 12.6 |
| Height (cm), mean ± SD | 150.6 ± 6.2 | 149.3 ± 5.6 | 150.6 ± 5.8 |
| BMI (kg/m²), mean ± SD | 24.5 ± 4.9 | 24.6 ± 5.2 | 24.9 ± 4.9 |
| MUAC (cm), mean ± SD | 28.0 ± 3.9 | 28.1 ± 4.3 | 28.4 ± 4.0 |
| **Haemoglobin, mean ± SD** |  |  |  |
| Automated analyser (g/dL) | 10.9 ± 1.3 | 11.2 ± 1.4 | 11.2 ± 1.2 |
| HemoCue 201+ (g/dL) | 10.5 ± 1.2 | 10.5 ± 1.2 | 10.5 ± 1.1 |
| **Iron and inflammatory biomarkers** | | | |
| Serum ferritin (µg/L), mean ± SD | 14.2 ± 8.1 | 14.6 ± 7.6 | 14.4 ± 7.4 |
| Serum ferritin, inflammation-adjusted (µg/L), mean ± SD | 13.5± 7.8 | 13.6 ± 7.5 | 13.8 ± 7.5 |
| AGP (mg/dL), mean ± SD | 71.7 ± 20.0 | 70.3 ± 20.7 | 78.5 ± 64.6 |
| AGP >100 mg/dL (1.0 g/L), % | 9.4 | 9.1 | 9.0 |
| CRP (mg/L), mean ± SD | 3.0 ± 3.5 | 2.5 ± 3.1 | 3.3 ± 4.3 |
| CRP >5.0 mg/L, % | 18.8 | 13.2 | 19.8 |
| **Inflammation stage, %** |  |  |  |
| No inflammation | 79.5 | 82.6 | 76.6 |
| Incubation | 11.1 | 8.2 | 14.4 |
| Early convalescence | 7.7 | 5.0 | 5.4 |
| Late convalescence | 1.7 | 4.1 | 3.6 |
| Hepcidin (ng/mL), mean ± SD | 3.7 ± 4.5 | 4.3 ± 5.7 | 3.9 ± 3.7 |
| Hepcidin <2.5 ng/mL, % | 58.1 | 50.4 | 49.6 |

*Data are mean ± SD or percentages, as indicated.*

*AGP = alpha-1-acid glycoprotein; BMI = body mass index; CRP = C-reactive protein; MUAC = mid-upper arm circumference; SD = standard deviation.*

*Ferritin concentrations were adjusted for inflammation using Thurnham et al.’s correction factors. Inflammation stages were defined as: no inflammation, CRP <=5 mg/L and AGP <=1.0 g/L; incubation, CRP >5 mg/L and AGP <=1.0 g/L; early convalescence, CRP >5 mg/L and AGP >1.0 g/L; and late convalescence, CRP <=5 mg/L and AGP >1.0 g/L.*

*The household wealth index was constructed using principal component analysis of household assets, drinking water source, and sanitation.*

*bLF 200 mg plus = 200 mg bovine lactoferrin plus 400 µg folic acid; bLF 400 mg plus = 400 mg bovine lactoferrin plus 400 µg folic acid; ferrous sulphate plus = 60 mg elemental iron as ferrous sulphate plus 400 µg folic acid.*

**Supplemental Figure S1: Average haemoglobin and ferritin concentrations over time by intervention group.**

*Mean haemoglobin and ferritin concentrations are shown at baseline, week 4, week 8, and week 12 for the three intervention groups. bLF 200 mg plus = 200 mg bovine lactoferrin plus 400 µg folic acid; bLF 400 mg plus = 400 mg bovine lactoferrin plus 400 µg folic acid; ferrous sulphate plus = 60 mg elemental iron as ferrous sulphate plus 400 µg folic acid.*

**Supplemental Table S2: Follow-up visit attendance by intervention group in the randomized population.**

| **Visit** | **All participants (N=555)** | **bLF 200 mg plus (n=186)** | **bLF 400 mg plus (n=185)** | **Ferrous sulphate plus (n=184)** | **p value** |
| --- | --- | --- | --- | --- | --- |
| Baseline | 547 (98.6) | 184 (98.9) | 181 (97.8) | 182 (98.9) | 1.000 |
| Visit 1 (week 4) | 418 (75.3) | 142 (76.3) | 142 (76.8) | 134 (72.8) | 0.547 |
| Visit 2 (week 8) | 372 (67.0) | 124 (66.7) | 126 (68.1) | 122 (66.3) | 0.890 |
| Visit 3 (week 12) | 349 (62.9) | 117 (62.9) | 121 (65.4) | 111 (60.3) | 0.595 |

*Data are n (% of randomized participants).*

*Visit 1, Visit 2, and Visit 3 correspond to weeks 4, 8, and 12 after treatment initiation, respectively.*

*p values are from Pearson chi-square tests comparing attendance proportions across the three intervention groups. bLF 200 mg plus = 200 mg bovine lactoferrin plus 400 µg folic acid;*

*bLF 400 mg plus = 400 mg bovine lactoferrin plus 400 µg folic acid; ferrous sulphate plus = 60 mg elemental iron as ferrous sulphate plus 400 µg folic acid.*

**Supplemental Table S3: Reasons for dropout by intervention group in the randomized population.**

| **Reason** | **bLF 200 mg plus**  **(n=186)** | **bLF 400 mg plus**  **(n=185)** | **Ferrous sulphate plus (n=184)** |
| --- | --- | --- | --- |
| Unwilling to take medicine | 28 (15.1) | 29 (15.7) | 34 (18.5) |
| Unwilling to give blood | 4 (2.2) | 6 (3.2) | 5 (2.7) |
| Could not be contacted / unavailable | 14 (7.5) | 13 (7.0) | 15 (8.2) |
| Migrated out of study area | 15 (8.1) | 9 (4.9) | 14 (7.6) |
| Reason not known | 8 (4.3) | 7 (3.8) | 5 (2.7) |
| Total | 69 (37.1) | 64 (34.6) | 73 (39.7) |

*Data are n (% of randomized participants). Percentages are calculated using the number randomized to each intervention group as the denominator.*

*bLF 200 mg plus = 200 mg bovine lactoferrin plus 400 µg folic acid; bLF 400 mg plus = 400 mg bovine lactoferrin plus 400 µg folic acid; ferrous sulphate plus = 60 mg elemental iron as ferrous sulphate plus 400 µg folic acid.*

**Supplemental Table S4: Logistic regression estimates for week 12 anaemia, iron deficiency, and iron-deficiency anaemia, adjusted for baseline status.**

| **Outcome at week 12** | **Ferrous sulphate plus** | **bLF 200 mg plus OR (95% CI)** | **p value** | **bLF 400 mg plus OR (95% CI)** | **p value** |
| --- | --- | --- | --- | --- | --- |
| Anaemia | Reference | 13.1 (6.4, 26.9) | <0.001 | 6.2 (3.3, 11.8) | <0.001 |
| Iron deficiency | Reference | 21.5 (10.9, 42.7) | <0.001 | 22.2 (11.2, 43.9) | <0.001 |
| Iron-deficiency anaemia | Reference | 33.1 (15.2, 72.2) | <0.001 | 23.1 (10.8, 49.6) | <0.001 |

*Odds ratios compare each bLF group with the ferrous sulphate plus group, which was the reference group. Models controlled for baseline status of the corresponding outcome.*

*bLF = bovine lactoferrin. bLF 200 mg plus = 200 mg bovine lactoferrin plus 400 µg folic acid; bLF 400 mg plus = 400 mg bovine lactoferrin plus 400 µg folic acid; ferrous sulphate plus = 60 mg elemental iron as ferrous sulphate plus 400 µg folic acid.*

**Supplemental Table S5: Estimated daily dietary iron intake from food consumption by intervention group.**

| **Measure** | **bLF 200 mg plus** | **bLF 400 mg plus** | **Ferrous sulphate plus** |
| --- | --- | --- | --- |
| Participants with dietary intake data, n | 178 | 173 | 173 |
| Iron consumption from food (mg/day), mean (SD) | 6.9 (3.3) | 7.26 (3.4) | 6.9 (3.5) |
| Low intake, <15 mg/day | 170 (95.5) | 167 (96.5) | 165 (94.8) |
| Appropriate intake, 15-18 mg/day | 7 (3.9) | 3 (1.7) | 7 (4.0) |
| High intake, >18 mg/day | 1 (0.5) | 3 (1.7) | 1 (0.5) |

*Data are mean (SD) or n (%).*

*Dietary iron intake was collected using a 7-day semi-quantitative food frequency questionnaire with standardized food photographs to estimate portion sizes. Daily dietary iron intake from food was estimated using the Bangladesh Food Composition Table, after converting cooked food weights to raw food weights and applying retention and yield factors. Values represent dietary iron from food and exclude iron supplied by trial supplements. Intake categories were defined as low (<15 mg/day), appropriate (15-18 mg/day), and high (>18 mg/day).*

*bLF = bovine lactoferrin. bLF 200 mg plus = 200 mg bovine lactoferrin plus 400 µg folic acid; bLF 400 mg plus = 400 mg bovine lactoferrin plus 400 µg folic acid; ferrous sulphate plus = 60 mg elemental iron as ferrous sulphate plus 400 µg folic acid.*

**Supplemental Figure S2: Risk-of-bias assessment for prior lactoferrin trials.**

*The figure summarizes risk of bias for prior lactoferrin trials and the current trial across random sequence generation/randomization process, allocation concealment/timing of identification or recruitment, blinding/deviations from intended interventions, missing outcome data/measurement of the outcome, selective reporting/selection of the reported result, and other bias. Green plus signs indicate low risk, yellow question marks indicate some concerns, and red minus signs indicate high risk.*
